# Comparison of Injuries Associated With Electric Scooters, Motorbikes, and Bicycles in France, 2019-2022

**DOI:** 10.1001/jamanetworkopen.2023.20960

**Published:** 2023-06-30

**Authors:** Arthur James, Anatole Harrois, Paer-Selim Abback, Jean Denis Moyer, Caroline Jeantrelle, Jean-Luc Hanouz, Mathieu Boutonnet, Thomas Geeraerts, Anne Godier, Julien Pottecher, Delphine Garrigue-Huet, Jean Cotte, Jean Pasqueron, Arnaud Foucrier, Tobias Gauss, Mathieu Raux

**Affiliations:** 1 Sorbonne University, GRC 29, AP-HP, DMU DREAM, Department of Anaesthesiology and Critical Care, Pitié-Salpêtrière Hospital, Paris, France; 2 Department of Anesthesiology and Critical Care, Bicêtre Hospital, AP-HP, University Paris Saclay, Le Kremlin Bicêtre, France; 3 Department of Anesthesiology and Critical Care Medicine, CHU Tours, Tours University Hospital, Tours, France; 4 Department of Anesthesiology and Critical Care Medicine, CHU Caen, Caen University Hospital, Caen, France; 5 Department of Anaesthesiology and Critical Care Medicine, Beaujon Hospital, DMU Parabol, AP-HP Nord, Université de Paris, Hospital Beaujon, Clichy, France; 6 Caen Normandy University, Unicaen, Caen, France; 7 Intensive Care Unit, Military Teaching Hospital Percy, Clamart, France; 8 Military Medical Academy, Val-de-Grâce, Paris, France; 9 Department of Anesthesiology and Critical Care, Toulouse University Hospital, University Toulouse 3 – Paul Sabatier, Toulouse, France; 10 Department of Anesthesia and Critical Care, Hôpital Européen Georges Pompidou, AP-HP, Université de Paris, Paris, France; 11 Hôpital de Hautepierre, Service d’Anesthésie, Réanimation & Médecine Péri-Opératoire - Université de Strasbourg, Faculté de Médecine, FMTS, EA3072, Hôpitaux Universitaires de Strasbourg, Strasbourg, France; 12 CHU Lille, Department of Anesthesiology and Critical Care, F-59000 Lille, France; 13 Intensive Care Unit, Military Teaching Hospital Sainte-Anne, Toulon, France; 14 Department of Anesthesiology and Critical Care, Hôpital Henri Mondor, Assistance Publique-Hôpitaux de Paris, Université Paris Est Créteil, France.; 15 Île-de-France Regional Health Agency, Paris, France; 16 Anaesthesia Critical Care, Grenoble Alpes University Hospital, 38700 Grenoble, France; 17 Sorbonne Université, INSERM, UMRS1158 Neurophysiologie Respiratoire Expérimentale et Clinique; AP-HP, Groupe Hospitalier Universitaire APHP-Sorbonne Université, site Pitié-Salpêtrière, Département d’Anesthésie Réanimation, F-75013 Paris, France

## Abstract

**Importance:**

Electric scooter (e-scooter) use is increasing in France and in many urban environments worldwide. Yet little is known about injuries associated with use of e-scooters.

**Objective:**

To describe characteristics and outcomes of major trauma involving e-scooters.

**Design, Setting, and Participants:**

A multicenter cohort study was conducted in France using the national major trauma registry between January 1, 2019, and December 20, 2022. All patients admitted to a participating major trauma center following a road traffic crash (RTC) involving an e-scooter, a bicycle, or a motorbike were included.

**Exposure:**

Included patients were compared according to the 3 mechanisms.

**Main Outcomes and Measures:**

The primary outcome was trauma severity as defined by the Injury Severity Score (ISS). Secondary outcomes included the trends of the number of patients per year, a comparison of the RTC epidemiologic factors, injury severity, resources used, and in-hospital outcomes.

**Results:**

A total of 5233 patients involved in RTCs were admitted (median age, 33 [IQR, 24-48] years; 4629 [88.5%] men; median ISS, 13 [IQR, 8-22]). The population included 229 e-scooter RTCs (4.4%), 4094 motorbike RTCs (78.2%), and 910 bicycle RTCs (17.4%). The number of patients treated following e-scooter RTCs increased by 2.8-fold in 4 years (from 31 in 2019 to 88 in 2022), while bicycle RTCs increased by 1.2-fold and motorbike RTCs decreased by 0.9-fold. At admission, 36.7% of e-scooter users had a blood alcohol content higher than the legal threshold (n = 84) and 22.5% wore a protective helmet (n = 32). Among e-scooter RTCs, 102 patients (45.5%) had an ISS of 16 or higher. This proportion was similar for patients with motorbike RTCs (1557 [39.7%]; *P* = .10) and bicycle RTCs (411 [47.3%]; *P* = .69). With a proportion of 25.9% (n = 50), patients with e-scooter RTCs had twice as many severe traumatic brain injuries (Glasgow Coma Scale ≤8) as motorbike RTCs (445 [11.8%]) and a proportion comparable to bicycle RTCs (174 [22.1%]). The mortality of e-scooter RTCs was 9.2% (n = 20), compared with 5.2% (n = 196) (*P* = .02) for motorbikes and 10.0% (n = 84) (*P* = .82) for bicycles.

**Conclusions and Relevance:**

The findings of this study suggest that trauma involving e-scooters in France has significantly increased over the past 4 years. These patients presented with injury profiles as severe as those of individuals who experienced bicycle or motorbike RTCs, with a higher proportion of severe traumatic brain injury.

## Key Points

**Question:**

What is the severity of injuries associated with electric scooters (e-scooters) compared with motorbikes or bicycles?

**Findings:**

In this cohort study of 5233 patients referred in a major trauma center, injuries due to e-scooters were as severe as those due to bicycle or motorbike crashes. The mortality associated with e-scooter road traffic crashes was 9.2%, compared with 10.0% for bicycles and 5.2% for motorbikes.

**Meaning:**

The findings of this study may inform health care professionals of the specificities of major trauma related to e-scooters and inform future e-scooter regulation decisions worldwide.

## Teaser

This cohort study compares the incidence and severity of trauma-related injuries in patients who experienced road traffic crashes while using electric scooters, motorbikes, and bicycles in France.

## Introduction

The French Academy of Medicine recently published a report^1^ on road traffic crashes (RTCs) involving electric scooters (e-scooters) in France. This report focused on injuries sustained by e-scooter users based on expert audits, scientific publications, national road safety reports, and press articles. The report nevertheless focused mainly on single-center case series, which did not allow for a precise description of patients with the most severe injuries.

Since 2012, the French Observatory for Major Trauma has developed the TraumaBase national registry. This registry includes all individuals with major trauma who are admitted to 1 of more than 20 major trauma centers and has collected data on nearly 50 000 patients. The participating major trauma centers are identified by the Regional Health Agencies as being within their respective geographic areas, the first-line resource for caring for individuals with the most severe injuries, whether in the context of road crashes, falls, assaults, or mass casualty situations.^2^

This registry collects descriptive data on the crash and prehospital and in-hospital evaluation data for all patients admitted after a severe trauma. This information allows for a very precise characterization of the person’s injuries and their effect on vital functions. The registry also includes information on the care provided, which gives an idea of the resources necessary for care at the patient, center, and regional level. In addition, the registry describes the in-hospital outcome of each patient, including survival and/or the occurrence of the most serious complications. This information is a valuable resource for describing the morbidity and mortality associated with severe trauma in France. Aware of the importance of e-scooters in public health, the registry has integrated the involvement of e-scooters in the mechanisms of crashes since 2019.

We therefore sought to provide original data from a large national registry to describe RTCs involving e-scooters. We especially hypothesized that these crashes result in injuries at least as severe as those caused by RTCs involving other vehicles, such as bicycles or motorbikes.

The objective of this work was to describe the epidemiologic characteristics of e-scooter RTCs over time in France; document the baseline characteristics of individuals with e-scooter injuries, their in-hospital management, and their outcomes; and compare them with those of patients with an RTC involving bicycles or motorbikes.

## Methods

### Study Design

We conducted a multicenter cohort study using data from the French National Trauma Registry (TraumaBase) from January 1, 2019, to December 20, 2022. A total of 26 trauma centers in France participated in the data collection. All data were collected as part of patient care, and no data were collected specifically for this project; data are deidentified. The TraumaBase registry has been approved by the Consultative Committee on the Processing of Health Research Information and the French National Commission on Informatics and Liberty. This study follows the Strengthening the Reporting of Observational Studies in Epidemiology (STROBE) reporting guideline.

### Population

The French trauma system implies that all injured patients are admitted to a trauma center based on a triage algorithm relying on information about the crash, as well as on the clinical presentation of the patient and their response to first-line treatment.^3,4^ After admission to a participating major trauma center, all consecutive patients were systematically included in the TraumaBase registry.^2,4,5^ In this study, all patients from the registry who experienced an RTC while using an e-scooter, a bicycle, or a motorbike were selected. We did not apply any exclusion criteria. Users of electric bicycles and electric motorbikes were categorized with bicycles and motorbikes.

### Data Management

We defined a severe traumatic brain injury as a Glasgow Coma Scale score of 8 or less at initial assessment.^6^ We defined hemodynamically unstable as any patient for whom at least 1 measured systolic blood pressure value was less than 100 mm Hg, who required the use of vasopressors or the transfusion of blood products before the computed tomography scan, or who needed transfusion of more than 4 units of packed red blood cells within the first 6 hours of admission. An Injury Severity Score (ISS) of 16 or higher was considered a marker of severe injuries.^7^

The Abbreviated Injury Scale (AIS) was used to describe each injury and its degree of severity (from 0 meaning no injury to 6 meaning unsurvivable injuries) within 6 body regions.^7^ An AIS score greater than or equal to 3 indicated the presence of at least 1 severe injury in the area of interest.

The ISS, when completed with information such as vital functions, age, and mechanism, allows for the calculation of the Trauma and Injury Severity Score (TRISS), which predicts mortality at the individual patient level and can thus provide a predicted mortality at a population level.^8^ The TRISS can be compared with the mean observed mortality at the population level as proposed by the American College of Surgeons Trauma Quality Improvement Program to determine excess mortality.^9^

The body topography of the injuries was characterized using the precise AIS codes used to capture each injury. From these AIS codes, all codes beginning with the number 7 were associated with upper extremity injuries and all codes beginning with the number 8 were associated with lower extremity injuries.^10^

### Outcomes

The primary outcome was the severity of trauma as defined by the ISS. Secondary outcomes included comparisons across the 3 groups of the trends of the number of patients per year, RTC epidemiologic factors (place of the crash, using a helmet, daytime or not, age, sex, American Society of Anesthesiologists physical status, blood alcohol content [BAC]), clinical and injury severity (unstable hemodynamic status, Glasgow Coma Scale score at baseline, Simplified Acute Physiology Score II, ISS, AIS of ≥3), in-hospital resources used (prehospital intubation, prehospital vasopressor use, hemorrhagic shock, surgery within the first 24 hours), and in-hospital outcomes (in-hospital death and causes, intensive care unit length of stay, and hospital length of stay).

### Comparison

We compared patients involved in e-scooter RTCs with bicycle and motorbike RTCs. This comparison was relevant because bicycles and motorbikes are typically included in studies involving patients who have experienced major trauma. Furthermore, all 3 devices involve falls from unenclosed 2-wheeled vehicles. These RTCs may or may not be preceded by a collision or followed by a crash.

### Statistical Analysis

Continuous variables are reported as medians (IQRs), while categorical variables are reported as numbers and their relative percentages. To compare the participants according to the mechanism (e-scooter, bicycle, or motorbike), χ^2^ tests were used for categorical variables and nonparametric Kruskal-Wallis tests were used for continuous variables.

Missing data were not imputed, except for the BAC, which was missing for nearly 30% of patients. To this end, a multiple imputation was conducted using variables that were found to be associated with the BAC in univariate analysis to complete the missing data.

A *P* value <.01 was considered significant to account for α risk inflation. All analyses were performed using R, version 1.4.1106 software (R Foundation for Statistical Computing).

## Results

### Epidemiologic Factors

A total of 20 172 patients were included in the TraumaBase registry; of these patients, 5233 (data available for 4629 men [88.5%]; 551 [10.5%] women; median age, 33 [IQR, 24-48] years; median ISS, 13 [IQR, 8-22]) met the inclusion criteria. Among these patients, 229 were treated following e-scooter RTCs, 4094 following motorbike RTCs, and 910 following bicycle RTCs. Among e-scooter RTCs, 161 patients (70.3%) were treated in 1 of the 6 trauma centers in the Paris urban region.

The annual number of RTCs was stable over time (Figure, A). Despite this apparent stability of RTC admissions, there was an increase in RTCs involving e-scooters (184%), a decrease in RTCs involving motorbikes (12%), and an increase in RTCs involving bicycles (24%) (Figure, B). Specifically, the number of patients treated in major trauma centers after an RTC involving an e-scooter increased from 31 to 88 per year over 4 years (Figure, C; eTable 1 in Supplement 1).

**Figure.**
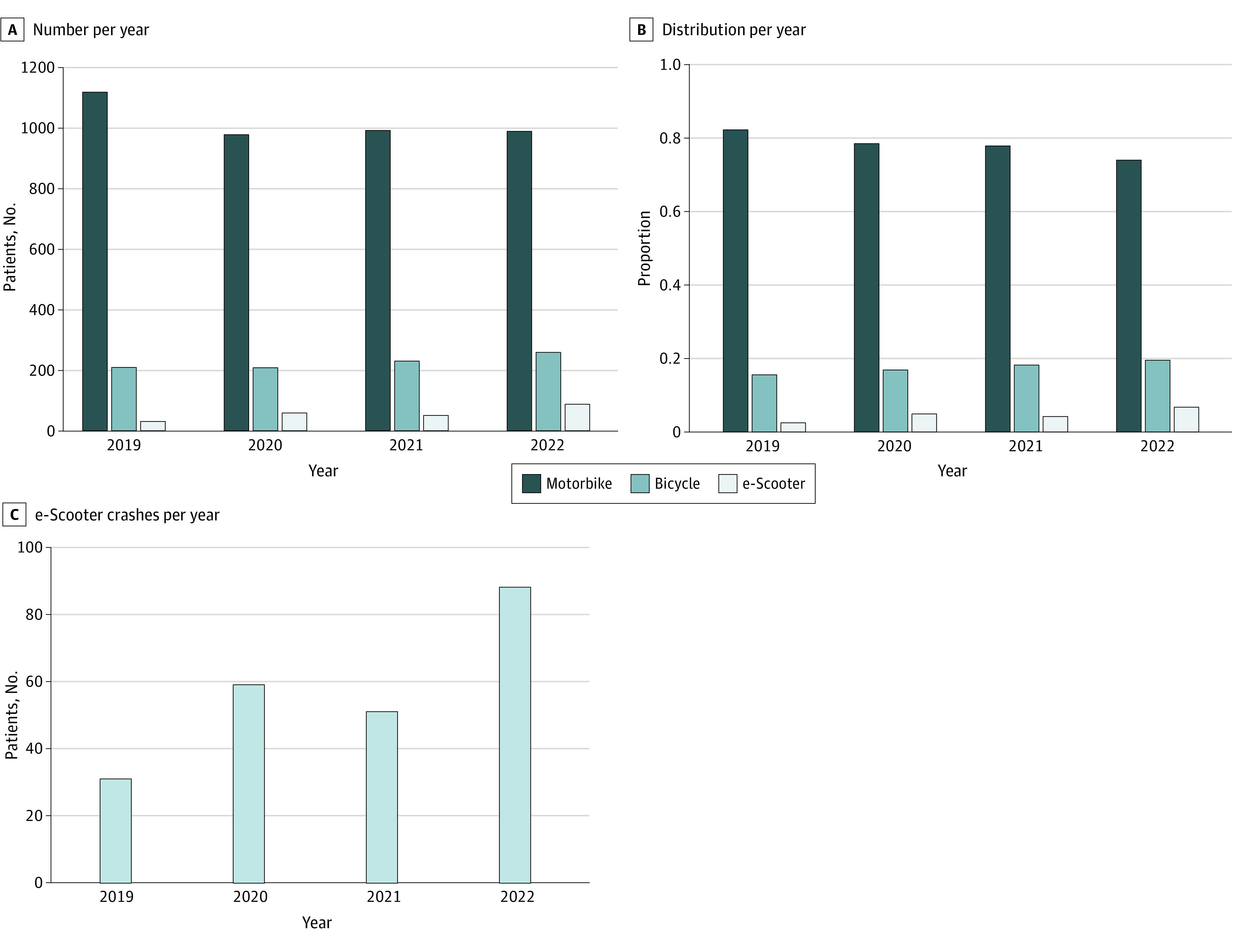
Four-Year Evolution of the Number of Patients Admitted in the Participating Major Trauma Centers

There was a fluctuation in the number of admissions for e-scooter RTCs both weekly (increased admissions on weekends; eFigure 1A in Supplement 1) and monthly (increased admissions during the summer months; eFigure 1B in Supplement 1). Two-thirds of cases of RTCs involving e-scooters (71.9%) and motorbikes (71.1%) occurred during shift periods (weekends and nights), while half of bicycle crashes (57.4%) occurred during shift periods.

### Population Description

Regarding individuals using e-scooters admitted to major trauma centers, 83% were men, and the median age was 33 (IQR, 25-46) years. This was similar to motorbike users, whose median age was 31 (IQR, 23-44) years, while bicycle users were significantly older, with a median age of 48 (IQR, 30-61) years (Table 1). Most of these patients (92.9%) did not have any comorbidities based on an American Society of Anesthesiologists physical status score less than or equal to 2. eTable 2 in Supplement 1 reports all information about missing data.

**Table 1.** Characteristics of the 5233 Patients Included in the Analysis

Variable	No. (%)				*P* value		
	Missing data	RTC					
		Motorbike (n = 4094)	Bicycle (n = 910)	e-Scooter (n = 229)	All groups	Motorbike vs e-Scooter	Bicycle vs e-Scooter
Age, median (IQR), y	14 (0.3)	31 (23-44)	48 (30-61)	33 (25-46)	<.001	.03	<.001
Sex							
Men	53 (1.0)	3726 (92.0)	713 (79.0)	190 (83.7)	<.001	<.001	.13
Women		324 (8.0)	190 (21.0)	37 (16.3)			
ASA-PS 1	59 (1.1)	3062 (75.6)	580 (64.6)	165 (72.4)	<.001	.53	.20
BAC >0.5 g/dL	1441 (27.5)	626 (20.7)	66 (10.8)	48 (28.9)	<.001	.02	<.001
Place of the crash							
Sport and recreational area	449 (8.6)	163 (4.3)	68 (8.5)	20 (9.3)	<.001	<.001	<.001
Transport area		3541 (94.0)	715 (89.2)	189 (87.5)			
Other		62 (1.7)	19 (2.3)	7 (3.2)			
Using a helmet	716 (13.6)	NA^a^	247 (49.3)	32 (22.5)	NA	NA	<.001
Crash occurring during daytime	28 (0.5)	1175 (28.9)	386 (42.6)	64 (28.1)	<.001	.86	<.001
Severity							
Unstable hemodynamic status	0	911 (22.3)	203 (22.3)	43 (18.8)	.46	.25	.28
GCS at baseline, median (IQR)	495 (9.5)	15 (14-15)	14 (10-15)	15 (8-15)	<.001	<.001	.34
Severe TBI	NA	445 (11.8)	174 (22.1)	50 (25.9)	<.001	<.001	.31
SAPS2	113 (2.2)	18 (11-28)	22 (15-37)	20 (13-33)	<.001	.009	.04
SOFA	133 (2.5)	1 (0-4)	1 (0-5)	1 (0-6)	<.001	.02	.51
ISS	220 (4.0)	12 (8-21)	13 (8-24)	13 (9-24)	.003	.07	.92
ISS ≥16	NA	1557 (39.7)	411 (47.3)	102 (45.5)	<.001	.10	.69
AIS ≥3							
Head	242 (4.6)	699 (17.9)	366 (42.2)	86 (38.4)	<.001	<.001	.34
Face	242 (4.6)	105 (2.7)	19 (2.2)	11 (4.9)	.08	.08	.05
Chest	242 (4.6)	1188 (30.5)	247 (28.5)	51 (22.8)	.03	.02	.10
Abdominal	242 (4.6)	514 (13.2)	71 (8.2)	30 (13.4)	<.001	>.99	.02
Extremities	242 (4.6)	1271 (32.6)	118 (13.6)	32 (14.3)	<.001	<.001	.88
External	242 (4.6)	2 (0.1)	1 (0.1)	0	.73	>.99	>.99
Extremity site							
Lower	242 (4.6)	2319 (56.6)	281 (30.9)	68 (29.7)	<.001	<.001	.79
Upper	242 (4.6)	876 (21.4)	217 (23.8)	39 (17.0)	.06	.14	.03
Both	242 (4.6)	435 (10.6)	68 (7.5)	13 (5.7)	.001	.02	.42

Abbreviations: AIS, Abbreviated Injury Scale; ASA-PS, American Society of Anesthesiologists–Physical Status; BAC, blood alcohol content; GCS, Glasgow Coma Scale; NA, not applicable; RTC, road traffic crash; SAPS2, Simplified Acute Physiology Score II; SOFA, sepsis-related organ failure assessment; TBI, traumtic brain injuries.

^a^ Helmet use data are not collected for motorbike RTCs in the TraumaBase.

The BAC at the time of admission was higher than the 0.5-g/dL threshold (legal level for an infraction in France) in 20.7% of motorbike (n = 626), 10.8% of bicycle (n = 66), and 28.9% (n = 48) of e-scooter users (*P* < .001). The sensitivity analysis, acknowledging missing data (27.5%) by using multiple imputation, highlighted that the BAC at the time of admission was above the 0.5-g/dL threshold in at least 22.5% (n = 923) of motorbike cases, 9.8% (n = 89) of bicycle cases, and 36.7% (n = 84) of e-scooter cases (Table 1; eTable 3 in Supplement 1).

Regarding helmet use at the time of the crash, individuals with e-scooter RTCs were half as likely as bicycle users to wear a helmet (22.5% [n = 247] vs 49.3% [n = 22]). Men wore a helmet in 24.8% (n = 28) of the crashes compared with 14.8% (n = 4) for women (*P* = .39). Age also appeared to be associated with helmet use, with helmets worn in 20.5% (n = 24) of the cases by those younger than 50 years and in 32.0% (n = 8) of those older than 50 years (*P* = .33). This analysis of helmet use was marked by a large proportion of missing data, with nearly 53% of data not reported.

### Baseline Severity

Initial hemodynamic instability was observed in 18.8% (n = 43) of patients with e-scooter RTCs, which did not differ significantly from that of patients with motorbike (911 [22.3%]) or bicycle (203 [22.3%]) RTCs (*P* = .46). The proportion of severe traumatic brain injuries among patients with e-scooter RTCs (50 [25.9%]) was significantly higher than in patients with motorbike RTCs (45 [11.8%]) and similar to that of patients with bicycle RTCs (17 [22.3%]) (both *P* < .001) (Table 1; eFigure 2 in Supplement 1).

### Injury Descriptions

With 45.5% of patients having an ISS greater than or equal to 16, patients with e-scooter RTCs had injuries as severe as those with motorbike RTCs (39.7%; *P* = .10) and bicycle RTCs (47.3%; *P* = .69) (Table 1). The analysis of the AIS scores showed that 58.5% (n = 131) of the patients with e-scooter RTCs had brain injuries of any severity, which was higher than for those with motorbike RTCs (1347 [34.5%]; *P* < .001) and similar to patients with bicycle RTCs (552 [63.7%]; *P* = .18) (Table 1). All 3 RTC groups presented with a median of 4 injuries per patient. Injuries to the lower extremities were more frequent than those to the upper extremities, regardless of the injury mechanism involved (Table 1).

### Resources Used

Prehospital intubation was required in 24.1% (n = 54) of patients with e-scooter RTCs compared with 17.8% (n = 714) for motorbike RTCs (*P* = .02) and 26.3% (n = 227) for bicycle RTCs (*P* = .57) (Table 2). The patients with e-scooter RTCs appeared to be at a lower risk of requiring more than 4 red blood cell transfusions in the first 6 hours, although the differences were not statistically significant, with a prevalence of 4.6% (n = 8) for e-scooter RTCs compared with 10.8% (n = 276) for motorbike RTCs (*P* = .01) and 8.4% (n = 50) for bicycle RTCs (*P* = .13). At 24 hours, 66.7% (n = 152) of the patients with e-scooter RTCs required at least 1 intervention compared with 73.7% (n = 2977) for motorbike RTCs and 55.7% (n = 501) for bicycle RTCs (Table 2).

**Table 2.** Resources Mobilized During Initial Management and In-Hospital Outcome 5233 Patients Included in the Analysis

Variable	No. (%)				*P* value		
	Missing data	RTC					
		Motorbike (n = 4094)	Bicycle (n = 910)	e-Scooter (n = 229)	All groups	Motorbike vs e-Scooter	Bicycle vs e-Scooter
Resource use							
Prehospital intubation	144 (2.8)	714 (17.8)	227 (26.3)	54 (24.1)	<.001	.02	.57
Prehospital vasopressor use	188 (3.6)	354 (9.9)	80 (9.4)	16 (7.1)	.57	.43	.36
Hemorrhagic shock	1893 (36.2)	276 (10.8)	50 (8.4)	8 (4.6)	.01	.01	.13
Surgery within the first 24 h	64 (1.2)	2977 (73.7)	501 (55.7)	152 (66.7)	<.001	.03	.004
Type of first surgery							
Orthopedic	1590 (30.4)	2219 (74.3)	154 (30.7)	43 (28.3)	<.001	<.001	.62
Neurologic		132 (4.4)	63 (12.8)	26 (17.1)			
Cardiothoracic		64 (2.1)	7 (1.4)	1 (0.7)			
ENT, maxillofacial, ophthalmic		79 (2.6)	17 (3.4)	2 (1.3)			
Spinal		22 (0.7)	5 (1.0)	1 (0.7)			
Endovascular		165 (5.5)	21 (4.2)	10 (6.6)			
Vascular		47 (1.6)	4 (0.8)	2 (1.3)			
Abdominal and urologic		133 (4.5)	16 (3.2)	5 (3.3)			
Not reported or other		129 (4.3)	214 (42.7)	62 (40.8)			
In-hospital outcomes							
Died	400 (7.6)	196 (5.2)	84 (10.0)	20 (9.2)	<.001	.02	.82
Cause of death							
TBI		75 (38.3)	53 (63.1)	13 (65.0)	<.001	.047	.42
Hemorrhagic shock		33 (16.8)	4 (4.8)	0			
MOF		38 (19.4)	8 (9.5)	3 (15.0)			
LWT		9 (4.6)	9 (10.7)	3 (15.0)			
Hypoxia		2 (1.0)	5 (6.0)	0			
Septic shock		2 (1.0)	0	0			
Other		22 (11.2)	5 (6.0)	0			
Unknown		15 (7.7)	0	1 (5.0)			
TRISS	1452 (28)	1 (1- 4)	2 (1-12)	2 (1-6)	<.001	<.001	.10
ICU length of stay, median (IQR), d	582 (11-1)	2 (1-6)	3 (2-7)	3 (2-6)	<.001	.03	.73
Hospital length of stay, median (IQR), d	670 (12-8)	8 (3-17)	7 (3-15)	6 (3-13)	.03	.06	.54

Abbreviations: ENT, ear, nose, and throat; ICU, intensive care unit; LWT, life-support withdrawal therapies; MOF, multiple organ failure; RTC, road traffic crash; TBI, traumatic brain injury; TRISS, Trauma and Injury Severity Score.

### In-Hospital Outcomes

The median intensive care unit lengths of stay were not clinically different across groups at 3 (IQR, 2-6) days for e-scooter RTCs, 2 (IQR, 1-6) days for motorbike RTCs, and 3 (IQR, 2-7) days for bicycle RTCs (*P* < .001). Likewise, in-hospital lengths of stay were 6 (IQR, 3-15) days for e-scooter RTCs, 8 (IQR, 3-17) days for motorbike RTCs, and 7 (IQR, 3-15) days for bicycle RTCs (*P* = .03) (Table 2).

The in-hospital mortality rate for e-scooter RTCs was 9.2% (n = 20), compared with 10.0% (n = 84) for bicycle RTCs (*P* = .82) and 5.2% (n = 196) for motorbike RTCs (*P* = .02). Traumatic brain injury was the leading cause of death for all 3 groups, accounting for 65.0% (n = 13) of deaths among e-scooter RTCs, 38.3% (n = 75) among motorbike RTCs, and 63.1% (n = 53) among bicycle RTCs (Table 2). The observed mortality did not differ significantly from the expected mortality (eFigure 3 in Supplement 1).

## Discussion

In this study, the number of individuals with e-scooter RTCs admitted to major trauma centers almost tripled over 4 years, while the number of those with bicycle RTCs increased by 24%, and motorbike RTCs decreased by 12%. These findings suggest that e-scooters are a source of serious crashes, with patients with e-scooter RTCs admitted to major trauma centers having injuries similar in severity to those with motorbike RTCs. These crashes seem to have a particular effect on the cranial sphere, which might be influenced by the frequency of risky behavior, such as not wearing a helmet or driving under the influence of alcohol. These injuries imply major use of in-hospital resources with, for example, more than half of the patients requiring surgery or a stay in the intensive care unit. In total, 10% of patients with e-scooter RTCs admitted to a major trauma center died during their hospital stay.

The French National Road Security Services indicated that 774 e-scooter RTCs of any injury severity occurred in 2020; furthermore, there was a 30% increase in the number of deaths and a 177% increase in the number of crashes compared with 2019.^11^ In 2021, Bagou et al^12^ published a study of 1186 patients with e-scooter RTCs admitted to an emergency department in France and highlighted that severe injuries were rare, with only 3.8% of all cases presenting with an AIS greater than or equal to 3. That study also acknowledged that patients with e-scooter RTCs had similar injuries compared with those with bicycle RTCs. The present study is thus intended to provide a major trauma center overview of e-scooter RTCs and report that e-scooter RTCs are also responsible for life-threatening injuries.^13,14^

The risk of craniofacial injuries is known for e-scooter RTCs,^14,15,16,17^ and this study confirms the low prevalence of helmet use among users.^12,18,19^ Wearing a helmet is strongly recommended for e-scooter users in France but is not mandatory. The French Academy of Medicine points out in its report that many stakeholders now support a change in regulation.^1^ Meanwhile, more places around the world are making it mandatory for e-scooter users to wear helmets. It is, for example, the case in Monaco; Brisbane, Australia; Los Angeles (for minors); and South Korea, while, at the opposite end of restrictions, Japan has lightened its regulations.

We could not confirm the predominance of upper extremity injuries compared with lower extremity injuries in our population of patients with major trauma, while several studies had raised this point.^15,18,20,21^ This report also points out that a significant proportion of patients with RTCs admitted to a major trauma center had consumed alcohol. These findings are in line with those presented in several single-center studies conducted in emergency departments in Europe.^12,19,21,22^

### Limitations

We acknowledge some potential limitations. First, the registry does not cover all centers in France, nor does it cover every hospital facility that admits trauma patients in each region (only major trauma centers). For these reasons, the undertriage rates for each population could not be reported and this study’s results should not be treated as an exhaustive picture of e-scooter RTCs in France, although it provides what is, to our knowledge, the largest description of patients with major trauma experiencing an e-scooter RTC reported in the literature. Similarly, we were unable to provide an aggregate count of the daily users of e-scooters within each trauma center area, as well as those involved in a crash, regardless of whether they sustained injuries during the inclusion period. However, a recent marketing study in France estimated that sales for e-scooters increased from €220 million (US $236 million) to €373 million (US $400 million) from 2019 to 2022 (70%) and the increase in the number of e-scooters for hire has already been linked to an increase in the number of hospital admissions.^23,24^

Second, it appears that individuals with e-scooter RTCs tend to have more severe injuries (according to the Glasgow Coma Scale, Simplified Acute Physiology Score, Sequential Organ Failure Assessment, and Revised Trauma Score) than those with motorbike RTCs. This could illustrate an overtriage of motorbike RTCs due to the frequent presence of a high kinetic criterion, which prompts prehospital physicians to refer these patients to a major trauma center even when the clinical assessment is reassuring. In contrast, patients with e-scooter RTCs, who are perceived as moving more slowly, do not benefit from this criterion and could therefore be at risk of undertriage. This underestimation of e-scooter RTCs was reported recently in an original study involving natural language processing conducted in Los Angeles.^25^

Third, the TraumaBase register does not collect some important information, such as vehicle speed, crash cause and description, socioeconomic status of the user, health status at the time of discharge (including the place of discharge and the autonomy), or long-term health consequences. These results, in conjunction with the findings of this study, could have been useful, especially within the context of possible changes in legislation regarding e-scooters in several countries. The question of long-term outcomes appears to be a great opportunity for development, especially given the fact that patients with major trauma appear to have a 3 times higher postdischarge 3-year mortality rate than other adults.^26^ This accompanies strong evidence that major trauma involves substantial long-term morbidity issues, with patients frequently developing problems such as chronic pain, difficulty walking, difficulty returning to work, or mental health impairment.^27,28^ The similarity between injuries encountered by patients with e-scooter RTCs and those of other RTCs suggests that these patients are very likely to be confronted with this burden.

## Conclusions

This cohort study provides insight from major trauma centers in France into the types of injuries associated with the growing use of e-scooters. It highlights a significant increase in the admission rate of patients with severe injuries due to e-scooter RTCs over the past 4 years. It also points out that these crashes appear to result in injuries as severe as those seen with bicycle or motorbike RTCs with a higher rate of severe traumatic brain injury and greater concern about risky behaviors. This study could provide meaningful information for stakeholders involved in regulating the use of these devices in France.

## Notes

Supplement 1. **eTable 1.** Annual Number of Patients Managed According To Injury Mechanism **eFigure 1.** Weekly and Monthly Fluctuation in the Number of Managed Patients **eTable 2.** Characteristics of the 5233 Patients Included in the Analysis **eTable 3.** Alcohol-Related Information **eFigure 2.** Distribution of Glasgow Scores According to the Different Mechanisms **eFigure 3.** Comparison Between Predicted and Observed Mortality According to Injury Mechanism

Supplement 2. **Nonauthor Collaborators.** French Observatory for Major Trauma (TraumaBase)

Supplement 3.
Data Sharing Statement
