# Supplementary material for: Comparison of Injuries Associated With Electric Scooters, Motorbikes, and Bicycles in France, 2019-2022
Source: JAMA Netw Open. 2023 Jun 30;6(6):e2320960. doi: 10.1001/jamanetworkopen.2023.20960 (PMC10314314; doi:10.1001/jamanetworkopen.2023.20960)
Supplement: Supplement 1. — eTable 1. Annual Number of Patients Managed According To Injury Mechanism eFigure 1. Weekly and Monthly Fluctuation in the Number of Managed Patients eTable 2. Characteristics of the 5233 Patients Included in the Analysis eTable 3. Alcohol-Related Information eFigure 2. Distribution of Glasgow Scores According to the Different Mechanisms eFigure 3. Comparison Between Predicted and Observed Mortality According to Injury Mechanism [file jamanetwopen-e2320960-s001.pdf]

Supplementary Online Content

James A, Harrois A, Abback PS, et al. Comparison of injuries associated with electric scooters, motorbikes, and bicycles in France, 2019-2022. *JAMA Netw Open*. 2023;6(6):e2320960. doi:10.1001/jamanetworkopen.2023.20960.

Table 1. Annual Number of Patients Managed According To Injury Mechanism

eFigure 1. Weekly and Monthly Fluctuation in the Number of Managed Patients

eTable 2. Characteristics of the 5233 Patients Included in the Analysis

eTable 3. Alcohol-Related Information

eFigure 2. Distribution of Glasgow Scores According to the Different Mechanisms

eFigure 3. Comparison Between Predicted and Observed Mortality According to Injury Mechanism

This supplementary material has been provided by the authors to give readers additional information about their work.

© 2023 James A et al. *JAMA Network Open.*

eTable 1. Annual Number of Patients Managed According To Injury Mechanism

	2019	2020	2021	2022
**Motorbikes RTC**	1123	982	996	993
**Bicycles RTC**	210	209	231	260
**eScooter RTC**	31	59	51	88

RTC, Road Traffic Crashes

© 2023 James A et al. *JAMA Network Open.*

eFigure 1. Weekly and monthly fluctuation in the number of managed patients

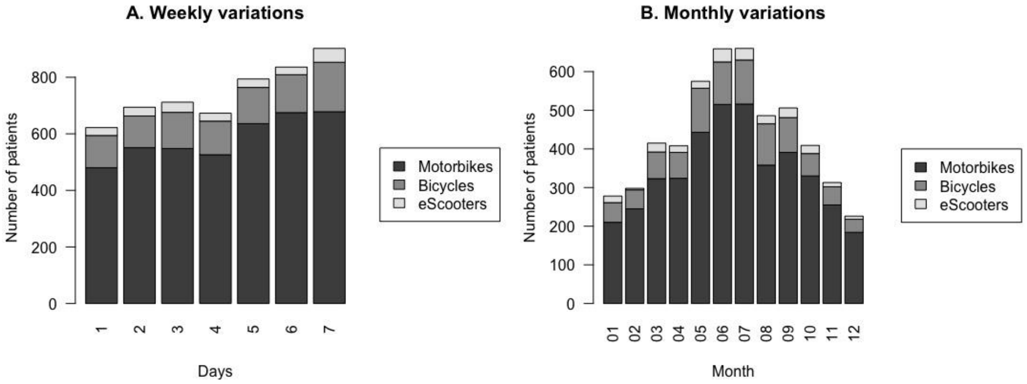

© 2023 James A et al. *JAMA Network Open.*

eTable 2. Characteristics of the 5233 patients included in the analysis

	**Overall missing data (n=5233)**	**Missing data in the motorbikes group (n=4094)**	**Missing data in the bicycles group (n=910)**	**Missing data in the eScooters group (n=229)**
Place of the crash	449 (8.6)	328 (8.0)	108 (11.9)	13 (5.7)
Using a helmet	716 (13.6)	-[£]	409 (44.9)	87 (38.0)
Crash occurring during daytime	31 (0.6)	23 (0.6)	4 (0.4)	1 (0.4)
Age (year)	14 (0.3)	11 (0.3)	3 (0.3)	0 (0)
Sex	53 (1.0)	44 (1.1)	7 (0.8)	2 (0.9)
ASA-PS=1	59 (1.1)	46 (1.1)	12 (1.3)	1 (0.4)
BAC (before imputation)	1441 (27.5)	1077 (26.3)	301 (33.1)	63 (27.5)
GCS at baseline	495 (9.5)	335 (8.2)	124 (13.6)	36 (15.7)
IGS	113 (2.2)	91 (2.2)	20 (2.2)	2 (0.9)
SOFA	133 (2.5)	107 (2.6)	24 (2.6)	2 (0.9)
ISS	220 (4.0)	174 (4.3)	41 (4.5)	5 (2.2)
AIS	242 (4.6)	194 (4.7)	43 (4.7)	5 (2.2)
Extremities injuries	275 (4.6)	194 (4.7)	43 (4.7)	5 (2.2)
Prehospital intubation, n (%)	144 (2.8)	335 (8.2)	46 (5.1)	5 (2.2)
Prehospital vasopressor use, n (%)	188 (3.6)	124 (3.0)	59 (6.5)	5 (2.2)
Hemorrhagic shock, n (%)	1893 (36.2)	1527 (37.3)	312 (34.3)	54 (23.6)
Surgery within the first 24 hours, n (%)	64 (1.2)	52 (1.3)	11 (1.2)	1 (0.4)
If yes-types of 1st surgery, n (%)	1590 (30.4)	1124 (27.0)	409 (44.9)	77 (33.6)
Died in hosp, n (%)	400 (7.6)	322 (7.9)	67 (7.4)	11 (4.8)
TRISS (%)	1452 (28)	1066 (26.0)	319 (35.1)	67 (29.3)
ICU length of stay, days	582 (11,1)	441 (10.8)	121 (13.3)	20 (8.7)
Hospital length of stay, days	670 (12,8)	502 (12.3)	149 (16.4)	19 (8.3)

Proportions are reported per columns. [£]Helmet use is not collected for motorbike patients in the TraumaBase

© 2023 James A et al. *JAMA Network Open.*

eTable 3. Alcohol-related information

		Missing data	Motorbikes (n=4094)	Bicycles (n=910)	eScooters (n=229)
Median BAC		1441 (27.5)	0.0 [0.0-0.1]	0.0 [0.0-0.1]	0.0 [0.0-1.1]
Patients with BAC > 0.5g/L		1441 (27.5)	626 (20.7)	66 (10.8)	48 (28.9)
Patients with BAC > 0.5g/L after imputation		0 (0.0)	923 (22.5)	89 (9.8)	84 (36.7)

BAC, Blood alcohol content. Multiple imputation involved age, sex, mechanism, baseline Glasgow score and baseline systolic blood pressure.

© 2023 James A et al. *JAMA Network Open.*

eFigure 2. Distribution of Glasgow scores according to the different mechanisms

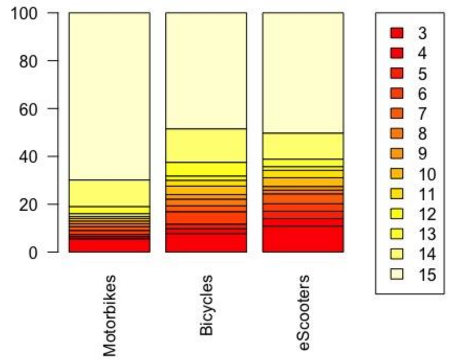

© 2023 James A et al. *JAMA Network Open.*

Supplementary material 6**:** eFigure 3. Comparison between predicted and observed mortality according to injury mechanism

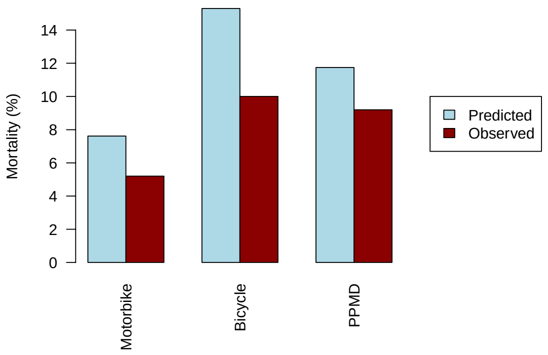

Mortality is predicted by the Trauma Injury Severity Score.

© 2023 James A et al. *JAMA Network Open.*
