# Supplementary material for: Comparison of Injuries Associated With Electric Scooters, Motorbikes, and Bicycles in France, 2019-2022
Source: JAMA Netw Open. 2023 Jun 30;6(6):e2320960. doi: 10.1001/jamanetworkopen.2023.20960 (PMC10314314; doi:10.1001/jamanetworkopen.2023.20960)
Supplement: Supplement 2. — Nonauthor Collaborators. French Observatory for Major Trauma (TraumaBase) [file jamanetwopen-e2320960-s002.pdf]

Supplemental Online Content: Nonauthor Collaborators
*First name, last name, and suffix (if applicable) are required and will appear in PubMed.

***Group Name(s): French Observatory for Major Trauma (TraumaBase)**

***First Name and Middle Initial(s)**	***Last Name**	***Suffix (eg, Jr, III)**	Academic Degrees	Institution	Location (city, state/province, country)	Role or Contribution, eg, chair, principal investigator	Group (if more than 1 Group listed in the byline) and/or Subgroup (eg, Steering Committee)
Gérard	Audibert		Pr	Nancy University Hospital	Nancy, France	Investigator	
Fanny	Bounes		Dr	Rangueil University Hospital	Toulouse, France	Investigator	
Thomas	Clavier		Dr	Rouen University Hospital	Rouen, France	Investigator	
Regis	Cloche		Dr	Valencienne General Hospital	Valencienne, France	Investigator	
Benjamin	Cohen		Dr	Tours University Hospital	Tours, France	Investigator	
Christophe	Couturier		Dr	Dunkerke General Hosptal	Dunkerque, France	Investigator	
Nathalie	Delhaye		Dr	HEGP University Hospital	Paris, France	Investigator	
Jacques	Duranteau		Pr	Bicêtre University Hospital	Kremlin-Bicêtre, France	Investigator	
Leslie	Dussau		Dr	Colmar Hospital	Colmar, France	Investigator	
Thierry	Floch		Dr	Reims University Hospital	Reims, France	Investigator	
Nicolas	Gatulle		Dr	Pitié-Salpétrière University Hospital	Paris, France	Investigator	
Sébastien	Gettes		Dr	Metz University Hospital	Metz, France	Investigator	
Pierre	Gosset		Dr	Amiens University Hospital	Amiens, France	Investigator	
Olivier	Langeron		Pr	Mondor University Hospital	Créteil, France	Investigator	
Marc	Leone		Pr	North University Hospital	Marseille, France	Investigator	
Eric	Meaudre		Pr	Toulon Army Hospital	Toulon, France	Investigator	
Marie	Moisan		Dr	Bordeaux University Hospital	Bordeaux, France	Investigator	
Jean	Pujo		Dr	Cayenne General Hospital	Cayenne, France	Investigator	
Véronique	Ramonda		Dr	Toulouse University Hospital	Toulouse, France	Investigator	
Julie	Rotival		Dr	Toulouse University Hospital	Toulouse, France	Investigator	
Marie	Werner		Dr	Bicêtre University Hospital	Kremlin-Bicêtre, France	Investigator	
Mathieu	Willig		Dr	Dijon University Hospital	Dijon, France	Investigator	
