# Supplementary material for: Comparison of Injuries Associated With Electric Scooters, Motorbikes, and Bicycles in France, 2019-2022
Source: JAMA Netw Open. 2023 Jun 30;6(6):e2320960. doi: 10.1001/jamanetworkopen.2023.20960 (PMC10314314; doi:10.1001/jamanetworkopen.2023.20960)
Supplement: Supplement 3. — Data Sharing Statement [file jamanetwopen-e2320960-s003.pdf]

Data Sharing Statement

James. Comparison of Injuries Associated With Electric Scooters, Motorbikes, and Bicycles in France, 2019-2022. *JAMA Netw Open*. Published June 30, 2023.
doi:10.1001/jamanetworkopen.2023.20960

Data
Data available: No

Additional Information
Explanation for why data not available: Relevant data can be available after reasonable request to the corresponding author and discussion with the working group.
